# Study of the Mechanical and Physical Behavior of Gypsum Boards with Plastic Cable Waste Aggregates and Their Application to Construction Panels

**DOI:** 10.3390/ma14092255

**Published:** 2021-04-27

**Authors:** Alejandra Vidales-Barriguete, Jaime Santa-Cruz-Astorqui, Carolina Piña-Ramírez, Marta Kosior-Kazberuk, Katarzyna Kalinowska-Wichrowska, Evangelina Atanes-Sánchez

**Affiliations:** 1 Departamento de Tecnología de la Edificación, Escuela Técnica Superior de Edificación, Universidad Politécnica de Madrid, 28040 Madrid, Spain; jaime.santacruz@upm.es; 2 Departamento de Construcciones Arquitectónicas y su Control, Escuela Técnica Superior de Edificación, Universidad Politécnica de Madrid, 28040 Madrid, Spain; carolina.pina@upm.es; 3 Faculty of Civil Engineering and Environmental Sciences, Bialystok University of Technology, 15-351 Bialystok, Poland; m.kosior@pb.edu.pl (M.K.-K.); k.kalinowska@pb.edu.pl (K.K.-W.); 4 Departamento de Ingeniería Mecánica, Química y de Diseño Industrial, Escuela Técnica Superior de Ingeniería y Diseño Industrial, Universidad Politécnica de Madrid, 28040 Madrid, Spain; evangelina.atanes@upm.es

**Keywords:** gypsum board, plastic waste, mechanical properties of gypsum board, physical properties of gypsum board, plaster applications

## Abstract

The objective of this study was to analyze the physico-mechanical properties of gypsum boards including plastic waste aggregates from cable recycling. The plastic cable waste is incorporated into the gypsum matrix without going through any type of selection and/or treatment, as it is obtained after the cable recycling process. In the experimental process, gypsum boards of different dimensions were manufactured and tested for their Young’s modulus, shock-impact resistance, flexural strength, thermal conductivity, and thermal comfort. The results obtained show a significant increase in the elasticity of the boards with plastic waste (limited cracking), compliance with the minimum value of flexural strength, and a slight improvement in the thermal conductivity coefficient (lower energy demand) and surface comfort (reduced condensation and greater adherence). Therefore, the analyzed material could provide a suitable alternative to currently marketed gypsum boards, contributing to sustainable construction not only in new constructions, but also in building renovations.

## 1. Introduction

In the construction sector, there has been a radical change in the manufacture of materials over the years. Where materials were once acquired locally and with minimal extraction and transformation processes, they can now be acquired anywhere in the world through laborious and sophisticated extraction and transformation processes [1].

Traditional materials, such as stone, gypsum, wood, and concrete, continue to be used however, in recent decades, alternative systems using lower environmental impact materials have been increasingly applied [2,3].

One of the most widespread materials in the world is gypsum board, mainly due to its ease and speed of assembly, versatility, and cleanliness. It has been widely used in residential buildings, offices, hotels, hospitals, and so on. Moreover, it is also used for the production of false ceilings, interior partitions, and wall linings. The boards are prefabricated (by gypsum manufacturing companies) and manufactured. Then, they are assembled on-site in a light metallic structure. This type of construction also called drywall puts less weight on the existing structures and offers a better level of comfort and greater ease of repair and/or modification when carrying out renovations [4].

At present, there is a trend for using materials from recycling, not only as alternatives to the natural resources, but also to avoid the accumulation of waste. This is why research in this field is essential, in order to achieve sustainable development and compliance with the guidelines and directives of the European Union.

One of the waste products that has generated the most social alarm is plastic. Around 350 million tons are manufactured per year around the world, and although it is true that the percentage of waste generated being recycled is increasing each year, an amount close to 40% still ends up in landfill [5]. Plastic is present in all areas of our lives due to its great diversity and the heterogeneity of its properties.

In the construction sector, plastics have been used in a wide variety of applications including pipes, window frames, insulation, coatings, floors, and cable coatings, among others. In the case of the latter, there are many factors that intervene in the requirement of one plastic or another in a cable, such as the type of atmosphere in which it is to be placed, the work stress that must be endured; the resistance to fire or flame spread, low smoke emissions, and so on. That is why these properties generate a wide range of possibilities, both in the current and future market for cables, with a multitude of plastic coatings, due to the heterogeneity mentioned above.

However, the recycling problems associated with plastic make it a recurring theme in the context of innovating materials, that is, trying to extend the life-cycle of plastic by reincorporating it into other materials [6]. Such studies have tried to improve acoustic insulation and for further lighten boards with the addition of expanded polystyrene (EPS) and cellulose solid waste [7,8,9,10]. Moreover, other types of waste have been incorporated, such as polypropylene and polyolefin waste which improve the resistance capacity against tensile stresses [11,12,13,14], the reducing effect of which has been verified, when greater length and quantity of fibers are incorporated; wood waste and ladle furnace slag, which improve thermal properties [15,16,17,18,19]; recycled paper, which improves the mechanical strength [20]; gypsum waste, which retains all its properties after several recycling processes [21,22,23,24,25]; polyurethane waste for its potential use in construction materials [26]; or polyvinyl alcohol, which increases the porosity and decreases the density of the compounds [27,28]. To the best or our knowledge, no studies have analyzed the effect of plastic cable waste in plaster matrices. In general, the studies mentioned above concerned finding ways to improve some of the properties of gypsum boards, but also to apply sustainability and circular economy criteria in these new “eco-products”.

This article presents the results of tests carried out on gypsum boards including aggregates of plastic cable waste, which were not subjected to any type of selection or previous treatment, despite their heterogeneity. The objective was to study the physico-mechanical properties of these boards and their potencial contribution to sustainable construction.

## 2. Materials and Methods

**Figure 1:**
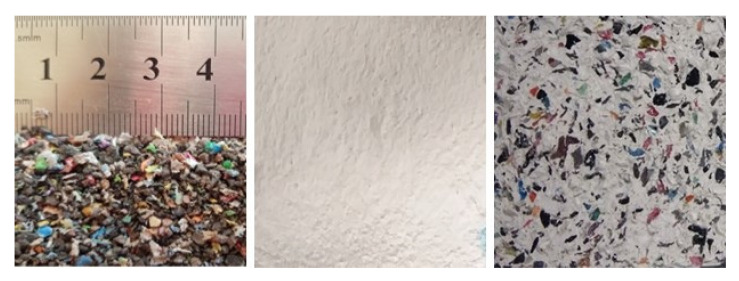
Left, plastic cable waste (PR). Center, sample without PR. Right, sample with PR.

A series of samples were prepared with Iberyola E-35 fine gypsum (type A) manufactured by Placo, water from the Canal de Isabel II in Madrid (Spain), and plastic cable waste provided by the recycling company Lyrsa Álava (Spain), with a maximum granulometric size of 3 mm and flat, rounded geometry (Figure 1). The water-to-gypsum mass ratio used was 0.8. The plastic cable waste, also referred to as PR (plastic residue) in this work was incorporated in percentages of 50, 60 and 70 into the gypsum mass (dosage in Table 1), with the intent of reusing the maximum amount of waste and also minimizing the use of raw material (gypsum and water). These series, with 3 samples each, were called E_0.8 50PR_, E_0.8 60PR,_ and E_0.8 70PR_, respectively. The minimum values for the properties of gypsum composites have been established in the UNE-EN 13279-1 standard [29].

In each test, a series of 3 samples without waste was made (a reference series called E_0.8_), against which the test results of the composites with plastic waste were compared. All the mixtures (Figure 1) were made according to the indications of the UNE-EN 13279-2 standard [30]. Prior to pouring water, the gypsum and polymeric waste were mixed manually for a few seconds, in order to prevent the waste from floating.

**Figure 2:**
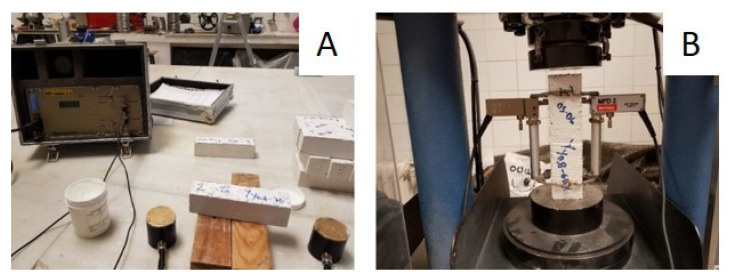
Young’s modulus test: (**A**) Dynamic test; and (**B**) static test.

Four series were prepared, including samples of different types, and five different properties were tested (Table 1):

Young’s modulus: Young’s modulus was determined by the dynamic and static methods according to the standard UNE-EN 13279-2 [30]. For the dynamic Young’s modulus, the ultrasonic propagation velocity was determined using the Ultrasonic tester E46 (Ibertest, Madrid, Spain). First, a gel was applied on each of the sides where the emitter and receiver were placed, in order to favor the transmission of the sound waves; passing time readings were taken longitudinally between these sides (Figure 2A). For the static Young’s modulus, the Autotest 200-10SW equipment was used (Ibertest, Madrid, Spain), on which the sample was positioned vertically and the extensometers were placed (Figure 2B). The equipment then compressed the sample and recorded the numerical value of Young’s modulus.

**Figure 3:**
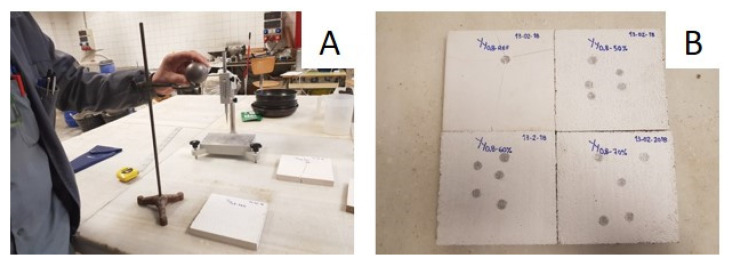
Shock-Impact Resistance test: (**A**) test equipment; and (**B**) board after test.

Shock-impact resistance: The shock-impact resistance was determined by carrying out tests to establish the hardness of the gypsum board surface, as indicated in the UNE-EN 520 standard [31]. Previously, Shore C hardness values were taken with a durometer (Baxlo, Barcelona, Spain), as specified in the UNE-102042 standard [32]. The samples were then placed on a rigid table face up. A steel ball, 50 mm in diameter and with 510 ± 10 g mass, was placed at a height of 500 ± 5 mm and was dropped (Figure 3). Finally, the diameter of the mark left by the impact was measured with 0.01 mm precision calipers. Each measurement was repeated five times and the mean value was calculated. The shock-impact resistance was measured by the depth of the mark determined by the steel ball after impact, according to the expression:

h = [D − √(D^2^ − d^2^)]/2 (1)

where, h is the depth of the mark after impact, in mm; D is the diameter of the steel ball, in mm; and d is the diameter of the mark after impact, in mm.

**Figure 4:**
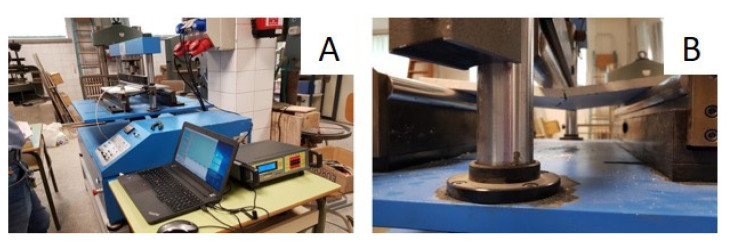
Flexural strength test: (**A**) test equipment; and (**B**) board with PR during test.

Flexural strength: The flexural strength was determined through testing the mechanical flexural strength of the boards subjected to a three-point bending flexural test, according to the UNE-EN 520:2004 standard [31]. The boards were placed horizontally on an ETI H0285 testing machine (Proeti, Madrid, Spain), equipped with two parallel cylindrical supports arranged on a leveled base (Figure 4). Then, by means of a loading roller, the load was applied continuously to the center of the board at a speed of approximately 200 N/min, until breakage. The data collection equipment was the MPX-22 (Pácam, Toledo, Spain) and the reading software used was EPO-1.

**Figure 5:**
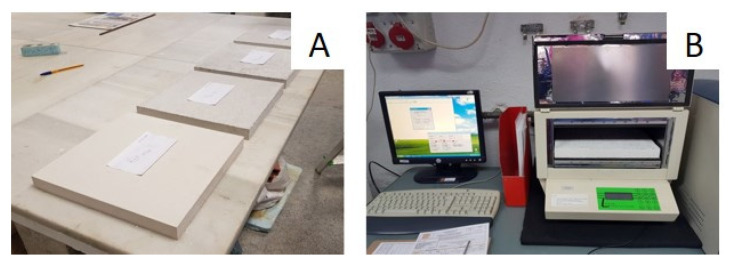
Thermal conductivity test: (**A**) The boards prepared for testing; and (**B**) board with PR during test.

Thermal conductivity: The thermal conductivity coefficient was determined using the “Determination of thermal resistance by means of guarded hot plate and heat flow meter methods. Products of high and medium thermal resistance” test defined in the UNE-EN 12667 standard [33]. For this purpose, LaserComp Fox 304 (TA Instruments, New Castle, UK) equipment was used, into which the previously weighed prepared samples were introduced (Figure 5). The equipment established, inside the samples, a constant ratio and uniformity in the heat flux density by means of a heating unit, heat flux meters, and a cooling unit. The equipment provided the following data: Apparent density, thermal conductivity, heat flux, and thermal resistance. For each composite, three samples were tested. The value presented in this work for each of the properties corresponds to their average value.

**Figure 6:**
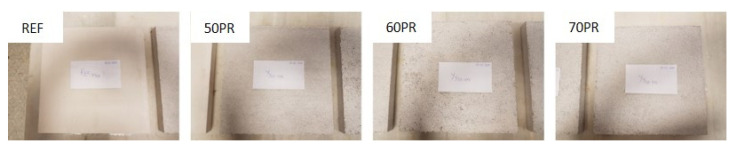
Boards used in the surface thermal comfort test.

Surface thermal comfort and visual aspect: The surface thermal comfort was determined using Villanueva’s Plaster Manual [34] (Figure 6). For this purpose, the heat penetration coefficient was calculated, which is given by the expression:

b = √(λ × c × ρ)(2)

where, b is the thermal penetration coefficient, in J/(s^0.5^m^2^K); λ is the thermal conductivity, in W/mK; c is the specific heat, in J/kgK, according to the Technical Building Code [35], for high hardness gypsum boards it is 1000 J/kgK; and ρ is the density, in kg/m^3^.

To evaluate the visual appearance of the composites, the boards were manually plastered and then painted.

## 3. Results and Discussion

The results obtained for the composites after performing the tests described above are shown below:

### 3.1. Young’s Modulus

**Figure 7:**
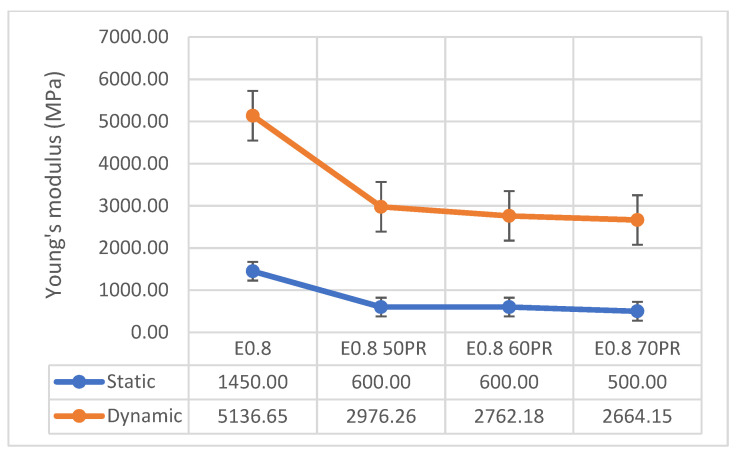
Young’s modulus values (UNE-EN 13279-2).

Figure 7 shows the Young’s modulus data obtained in the two variants studied: Static and dynamic. These tests were carried out on Series I composites.

The static Young’s modulus showed a significant decrease in the stiffness of all the composites, with respect to the reference sample. Specifically, the composites with 50% and 60% PR were 58.62% more elastic, and the composite with 70% PR was 65.52% more elastic, which shows the effect of plastic waste on the elastic behavior of the material. Accordingly, the most elastic composite containing PR was E_0.8 70PR_ (500.00 MPa) and the stiffest were both E_0.8 50PR_ and E_0.8 60PR_ (600.00 MPa).

The results for the dynamic Young’s modulus also indicated a significant decrease in the stiffness of the mixtures with respect to the reference sample, close to 50%. The composite with 50% PR decreased the dynamic Young’s modulus by 42.04%, the composite with 60% PR by 46.23%, and the composite with 70% PR by 48.13%. In this case, the most elastic PR composite was also E_0.8 70PR_ (2664.15 MPa) and the stiffest was E_0.8 50PR_ (2976.26 MPa). These data corroborate the results obtained in other studies using gypsum and rubber waste [36,37,38,39], in which the elasticity of the compounds significantly increased with the content of polymer waste.

### 3.2. Shock-Impact Resistance

Considering that gypsum boards can be exposed to impact from objects at any time, the resistance of the composites in this regard was tested. In the analysis of the shock-impact resistance test, carried out in Series II, the diameter and depth of the steel ball mark were determined, as shown in Table 2.

In the case of the reference board E_0.8_, the first impact broke the sample. However, in all the impacts on the samples with PR, the steel ball rebounded, which corroborated the increase in elasticity achieved in the composites under study (verified and analyzed with Young’s modulus in the previous section), showing the composite E_0.8 50PR_ the highest shock-impact resistance (0.73 mm mark depth). These results are also similar to those obtained by other researchers [36,37,40,41], in which the improvement of the shock-impact resistance capacity was observed in mixtures of plaster with polymeric residues.

### 3.3. Flexural Strength

Considering that the boards can work under the mechanical effect of bending, tests for flexural strength were carried out on the composites. Table 3 shows the results of the flexural breaking load and the displacement of the boards made with the Series III mixtures.

**Figure 8:**
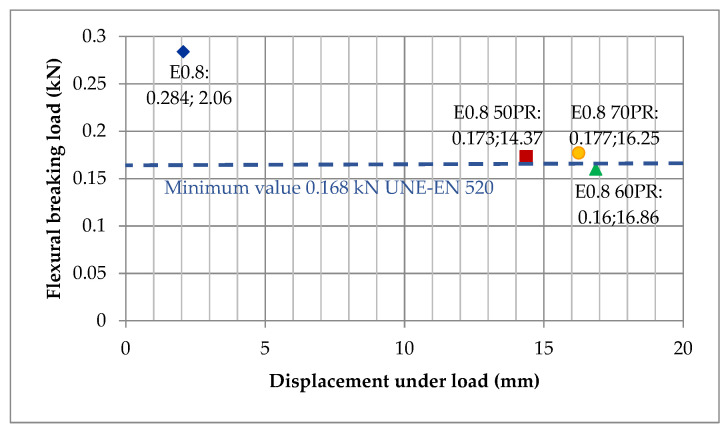
Average load-displacement values of the composites (UNE-EN 520).

The mean flexural breaking load in the mixtures decreased by 39.08%, 43.66% and 37.68% respectively, with 50%PR, 60%PR and 70%PR content, compared to the reference sample. A similar phenomenon occurred in the flexural tests of prismatic samples described in [42]. In that article, SEM images showed, that the dihydrate gypsum crystals form a porous network that surrounds the much larger particles of plastic waste. These images showed the presence of a certain amount of discontinuities in the gypsum-pellet junction, which would explain the decrease in the mechanical properties of these compounds. On the other hand, the displacement admitted by the boards of the composites with 50%PR, 60%PR and 70%PR exceeded the displacement admitted by the reference board by 697.57%, 818.45% and 788.83%, respectively. This verifies, once again, the results obtained in the Young’s modulus tests, in which the elasticity of the composites with PR increased considerably. All of the boards, except the E_0.8 60PR_ board, exceeded the minimum breaking load value (Figure 8) required by the UNE-EN 520 standard (0.168 kN); however, it is true that, in this standard, the boards were prepared with a sheet of paper or cardboard on one of their sides, which was not taken into account in the case of the boards tested. Therefore, it would be advisable to carry out the test with boards prepared with a covering layer of either paper or cardboard and recheck their flexural breaking load value, which would possibly sufficiently increase the E_0.8 60PR_ board qualities to meet the requirements of the aforementioned standard. The PR composite with the highest flexural strength among the boards was E_0.8 70PR_ (0.177 kN) and the one with the lowest flexural strength was E_0.8 60PR_ (0.160 kN).

**Figure 9:**
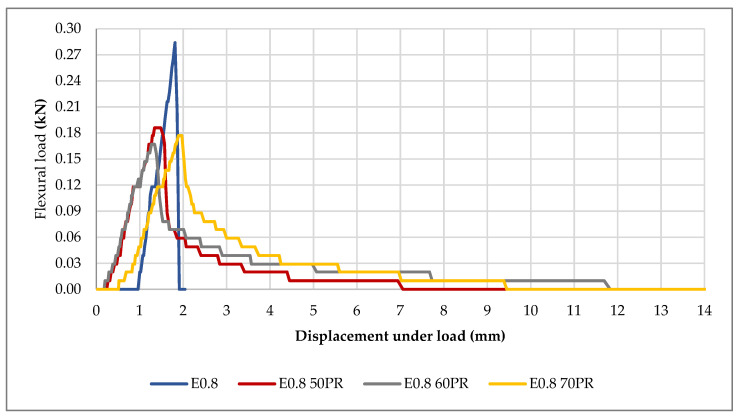
Load-displacement of the composites.

From the load-displacement graphs of each of the mixtures, the higher flexural strength and stiffness of the reference, as well as the reduction of the flexural strength of the composites with PR and their higher deformation capacity (elasticity), can be deduced (Figure 9).

Similar results have also been observed in the studies of other researchers [14,40,41,43] where, by introducing polymeric residues, the deforming capacity of the mixtures was increased.

### 3.4. Thermal Conductivity

The results obtained after the test according to the UNE-EN 12667, carried out in Series IV, are shown in Table 4. Density, thermal conductivity, heat flux, and thermal resistance were determined.

Compared to the reference sample, the apparent density of the samples increased with the incorporation of PR and, a higher content of pellets. This increase was in agreement with the evolution of porosity of these samples, as determined in previous studies [44], with a pore volume value of 0.5419 cm^3^/g for the reference sample. This value progressively decreased to values of 0.467, 0.4276, and 0.3447 cm^3^/g for the composites with 50%, 60%, and 70% PR, respectively, resulting in decreases in porosity of 22.18%, 20.57%, and 32.58%, respectively, compared to the reference sample. In other words, the incorporation of PR produced a lower porosity, which resulted in a higher value for the apparent density of the composites with PR, compared to the reference one; the higher the PR content, the higher the density, as the pore volume decreased.

The value obtained with this method for the thermal conductivity of the reference sample was very similar to the generic value of 0.25 W/mK assigned in the Technical Building Code [45] for gypsum boards.

In none of the three thermal properties in Table 4 (thermal conductivity, heat flux, and thermal resistance) was an increasing or decreasing linear relationship observed between the mixtures with PR and the reference. In the composites with 50% PR and 60% PR, the thermal conductivity values decreased by 5.97% and 7.36%, respectively. Consequently, the heat flux decreased, by 5.27% and 9.75%, and the thermal resistance value increased by 5.20% and 12.22%, respectively. However, in case of the composite with 70% PR, the thermal conductivity increased (by 1.02%) with respect to the reference, the heat flux decreased (by 1.84%), and the thermal resistance also slightly increased (by 1.51%). These results concerning the thermal properties depending on the PR content in the samples can be explained on the basis of two factors with opposite effects on these properties. Taking into account the lower porosity and, therefore, the lower presence of air in pores, the thermal conductivity of the composites with PR should be higher than that of the reference, where a higher amount of PR should lead to a higher thermal conductivity. However, according to the results presented in Table 4, this was not the case. This behavior may have been due to the fact that the thermal conductivity was affected not only by the pore volume of the samples but also by the presence of the pellets, which make their own contribution to the thermal conductivity of the composites.

The most abundant polymers in electric cable waste are PVC and PE [46], which have exhibited conductivities of 0.17 W/(mK) for PVC and 0.5 and 0. 33 W/(mK) for high and low-density PE, respectively [45]. The pellets used in this work were a heterogeneous mixture of polymers, and it is possible that the thermal conductivity of PR may have been lower than that of gypsum; as such, the presence of PR in the composites would have led to a decrease in the thermal conductivity of the samples. This would have been especially the case with samples with higher PR content, as was observed in the samples with 50% and 60% PR having values lower than the reference, and even lower for the sample with higher pellet content (E0.8_60PR_).

For sample E0.8_70PR_, this decrease in thermal conductivity due to the high PR content (70%) would have been counteracted by a pore volume much lower than the pore volumes of the rest of the samples, which would have globally led to an increase in thermal conductivity to a value slightly higher than that of the reference sample. However, the values of the three thermal properties in Table 4 for the E0.8_70PR_ compound were very similar to the values obtained in the reference, with a difference of less than 2% in all of them. Therefore, with regards to the thermal properties according to the PR content, at concentrations of up to 60% PR, the effect of the lower conductivity of the pellets with respect to the gypsum prevailed, resulting in lower values for the thermal conductivity of the composites, compared to the reference. At high concentrations (e.g., 70% PR), the effect of a much lower pore volume prevailed, which increased the thermal conductivity of the composite to a value close to that of the reference.

The composite with the highest apparent density, E0.8_70PR_, had the highest thermal conductivity coefficient (0.2469 W/mK), the highest heat flux (149.0 W/m^2^), and the lowest thermal resistance (0.1346 m^2^K/W). However, the composite with the lowest apparent density, E0.8_50PR_, did not present the lowest conductivity coefficient, lowest heat flux, or highest thermal resistance; rather, it was the intermediate density composite, E0.8_60PR_, with values of 0.2264 W/mK, 137.0 W/m^2^, and 0.1488 m^2^K/W, respectively.

In any case, the results of Table 4 indicate that the thermal behavior of the composites with PR did not present great differences, with respect to the gypsum reference. The incorporation of pellets up to 60% of PR did not imply a high increase in the insulation capacity, as the thermal conductivity coefficient of the materials studied was much higher with respect to the thermal conductivity coefficient of an insulating material, such as expanded polystyrene or mineral wool, with thermal conductivities of 0.039 W/(mK) and 0.050 W/(mK) respectively. However, it should be noted that, according to the data obtained, both the E0.8_50PR_ and E0.8_60PR_ boards showed a slight thermal improvements with respect to the board without PR, which makes them more energy-efficient than the traditional material, corroborating the studies of other researchers using polymeric waste [36,47,48,49].

### 3.5. Surface Thermal Comfort and Visual Appearance

The thermal conductivity coefficients and densities obtained in the thermal test “Determination of thermal resistance by means of guarded hot plate and heat flow meter methods. Products of high and medium thermal resistance”, defined in the UNE-EN 12667 standard (Table 4), are used to determine the values of thermal comfort or perceived sensation when touching the surface of a material. With these data, the heat penetration coefficient of each of the composites was calculated (Table 5): The lower this coefficient is, the more comfortable the surface of the material is to the touch, the less cold it is and, therefore, the less condensation that may appear on it [34].

The surface comfort of all the composites, expressed in terms of the coefficient of thermal penetration, was very similar to that of the reference sample. The mixtures with 50%PR and 60%PR showed a slight improvement (of about 2.50%), while the mixture with 70%PR slightly worsened (by 2%), due to the slight increase in its coefficient of thermal conductivity, as analyzed in the previous section. The composite with the lowest heat penetration coefficient and, therefore, the one that had the best surface comfort was E_0.8 60PR_ (474.45 J/(s^0.5^m^2^K)); however, it had a value very similar to that of E_0.8 50PR_. The composite with the highest heat penetration coefficient and, therefore, the one that had the worst surface comfort was E_0,8 70PR_ (496,56 J/s^1/2^m^2^K).

**Figure 10:**
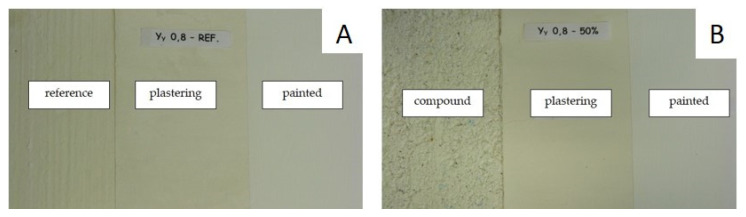
Board finishes: (**A**) Reference board; an (**B**) composite board with PR.

Generally, such boards are treated with a surface finish that can be affected by small settlement movements or can expand due to temperature changes in the building. Figure 10A shows images of the reference composite (left image), with gypsum plastering (middle image), and after paint application (right image). Figure 10B shows the same images for a composite with PR. The visual effect of the surface finishes of the composites with PR (Series IV), once the paint had been applied (Figure 10B), was similar to that for the composites without PR (Figure 10A). An improvement in the behavior of the composites with PR, with respect to the appearance of cracks, is highlighted; this was due to their high elasticity, as observed in the flexural strength test. The fact that the composites with PR without any coating have a rough appearance, as shown in the image on the left of Figure 10B, is also an advantage, as this facilitates the adhesion of coating materials such as paints or tiling.

## 4. Conclusions

Boards including plastic cable waste showed a significant improvement, in terms of cracking and impact resistance, compared to traditional boards. This was because the incorporation of the plastic cable waste in the gypsum matrix increased elasticity by approximately 50%, compared to the reference composites without added waste.

In terms of flexural breaking load, all the composites exceeded the minimum value established in the standards, except the one with 60%PR, which had a value of 0.008 kN lower than said minimum value. It should be noted that the composites were not manufactured with a paper or cardboard coating on one of the sides (as indicated in the standard), which suggests that, if this were the case, the flexural strength value would increase, thus potentially complying with the standard.

The thermal behavior of the composites with PR did not show major differences, with respect to the gypsum reference composite; however, the thermal properties also improved slightly with pellet contents of up to 60%, which led to a contribution to a lower energy demand, with respect to the traditional material.

The same was true for the finish of the composites which, although similar to that of the reference, showed a slight improvement in surface comfort in the composites with plastic cable waste. This means that the boards were less cold, and therefore, less condensation may appear on them. Their roughness, before any finishing, also indicated an increase in the adherence to the support or to the final finish.

For all these reasons, it is considered that the use of plastic cable waste as aggregates for the manufacture of gypsum boards provides advantages over traditional boards. It would be of interest to use them not only in new construction, but also in building renovations, where the appearance of cracks, condensation, breakage, and/or spalling due to impact, or detachment of the finishing material (e.g., paint or tiling) would be reduced.

In addition, the manufacturing of this type of board would serve as a contribution to sustainable construction, not only due to the improvement in the thermal properties of the board (lower energy demand), but also because of the incorporation of plastic waste as a secondary raw material, which minimizes this type of waste and reduces the amount of gypsum and water used for the manufacture of the boards.

## Figures and Tables

**Table 1:** Name of samples, composition, dimensions, and test performed.

Series	Name	PR Pellets (wt%) *	Water-to Gypsum Ratio	Dimensions ** (cm)	Test Performed
I	E_0.8_	0%	0.8	16 × 4 × 4	Young’s modulus
	E_0.8 50PR_	50%			
	E_0.8 60PR_	60%			
	E_0.8 70PR_	70%			
II	E_0.8_	0%	0.8	15 × 15 × 2	Shock-Impact Resistance
	E_0.8 50PR_	50%			
	E_0.8 60PR_	60%			
	E_0.8 70PR_	70%			
III	E_0.8_	0%	0.8	40 × 30 × 1	Flexural strength
	E_0.8 50PR_	50%			
	E_0.8 60PR_	60%			
	E_0.8 70PR_	70%			
IV	E_0.8_	0%	0.8	30 × 30 × 3	Thermal conductivity Surface thermal comfort
	E_0.8 50PR_	50%			
	E_0.8 60PR_	60%			
	E_0.8 70PR_	70%			

* Calculated on the mass of gypsum. ** Length × Width × Height.

**Table 2:** Shock-impact resistance (UNE-EN 520).

Series	Name	Shore C Hardness	Mark Diameter d (mm)	Mark Depth h (mm)
II	E_0.8_	78	14.70 ± 0.40	1.17 ± 0.06
	E_0.8 50PR_	81.17	11.68 ± 0.97	0.73 ± 0.11
	E_0.8 60PR_	80.6	13.70 ± 0.97	1.01 ± 0.14
	E_0.8 70PR_	81.6	13.18 ± 0.63	0.93 ± 0.09

**Table 3:** Flexural breaking load of boards and displacement under load (UNE-EN 520).

Series	Name	Flexural Breaking Load (kN)	Displacement under Load (mm)
III	E_0.8_	0.284 ± 0.02	2.06 ± 0.49
	E_0.8 50PR_	0.173 ± 0.03	14.37 ± 1.22
	E_0.8 60PR_	0.160 ± 0.02	16.86 ± 0.69
	E_0.8 70PR_	0.177 ± 0.01	16.25 ± 0.79

**Table 4:** Apparent density, thermal conductivity, heat flux, and thermal resistance (UNE-EN 12667).

Series	Name	Density (kg/m^3^)	Thermal Conductivity—λ (W/mK)	Heat Flux (W/m^2^)	Thermal Resistance (m^2^K/W)
IV	E_0.8_	969.97	0.2444 ± 0.02	151.8	0.1326
	E_0.8 50PR_	980.3	0.2298 ± 0.01	143.8	0.1395
	E_0.8 60PR_	994.27	0.2264 ± 0.03	137	0.1488
	E_0.8 70PR_	998.67	0.2469 ± 0.02	149	0.1346

**Table 5:** Thermal comfort due to heat penetration coefficient.

Series	Name	Specific Heat (J/kgK)	Heat Penetration Coefficient (J/(s^0.5^m^2^K))
IV	E_0.8_	1000	486.89
	E_0.8 50PR_	1000	474.63
	E_0.8 60PR_	1000	474.45
	E_0.8 70PR_	1000	496.56

## Data Availability

Data sharing is not applicable to this article.

## References

[1] Vidales Barriguete A. (2019). Caracterización Fisicoquímica y Aplicaciones de Yeso con Adición de Residuo Plástico de Cables Mediante Criterios de Economía Circular. Ph.D. Thesis.

[2] Ramírez C.P., Merino M.D.R., Arrebola C.V., Barriguete A.V., Kosior-Kazberuk M. (2019). Analysis of the mechanical behaviour of the cement mortars with additives of mineral wool fibres from recycling of CDW. Constr. Build. Mater..

[3] Kosior-Kazberuk M., Krassowska J., Barriguete A.V., Rodríguez C.P. (2018). Parámetros de la Fractura del Hormigón Reforzado con Fibras de Basalto = Fracture Parameters of Basalt Fiber Reinforced Concrete. An. Edif..

[4] Quinchía Figueroa A.M., Valencia García M.F., Giraldo Orozco J.M. (2007). Uso de lodos provenientes de la industria papelera en la elaboración de paneles prefabricados para la construcción. Rev. EIA.

[5] PlasticsEurope Plásticos—Situación en 2018. https://www.plasticseurope.org/es/resources/publications/1240-plasticos-situacion-en-2018.

[6] Arandes J.M., Bilbao J., López Valerio D. (2004). Reciclado de residuos plásticos. Rev. Iberoam. Polímeros.

[7] De Oliveira K.A., Barbosa J.C., Christoforo A.L., Molina J.C., Oliveira C.A.B., Bertolini M.S., Gava M., Ventorim G. (2019). Sound absorption of recycled gypsum matrix composites with residual cellulosic pulp and expanded polystyrene. Bioresources.

[8] San-Antonio-González A., Merino M.D.R., Arrebola C.V., Villoria-Sáez P. (2016). Lightweight Material Made with Gypsum and EPS Waste with Enhanced Mechanical Strength. J. Mater. Civ. Eng..

[9] Muñoz Muñoz D.R., Narváez Pupiales J.I. (2019). Construcción Sostenible a Partir de Paneles Prefabricados Utilizando yeso y Celulosa Reciclada. Bachelor’s Thesis.

[10] Merino M.D.R., Sáez P.V., Longobardi I., Astorqui J.S.C., Porras-Amores C. (2019). Redesigning lightweight gypsum with mixes of polystyrene waste from construction and demolition waste. J. Clean. Prod..

[11] Suárez F., Felipe-Sesé L., Díaz F.A., Gálvez J.C., Alberti M.G. (2019). Comportamiento a fractura de yeso con adición de fibras poliméricas. An. Mecánica Fract..

[12] Silva Collado D. (2019). Propuesta de Paneles Prefabricados Para Particiones Interiores con Compuesto de yeso Reforzado con Fibras de Polipropileno Obtenidas de Residuos de Toallitas Húmedas. Bachelor´s Thesis.

[13] Deng Y.-H., Furuno T. (2001). Properties of gypsum particleboard reinforced with polypropylene fibers. J. Wood Sci..

[14] Mohandesi J.A., Sangghaleh A., Nazari A., Pourjavad N. (2011). Analytical modeling of strength in randomly oriented PP and PPTA short fiber reinforced gypsum composites. Comput. Mater. Sci..

[15] Morales-Conde M., Rodríguez-Liñán C., Pedreño-Rojas M. (2016). Physical and mechanical properties of wood-gypsum composites from demolition material in rehabilitation works. Constr. Build. Mater..

[16] Alonso Á., Gadea J., Gutiérrez-González S., Calderón V. (2019). Impact of Plasterboard with Ladle Furnace Slag on Fire Reaction and termal Behavior. Fire Technol..

[17] Pedreño-Rojas M., Morales-Conde M., Pérez-Gálvez F., Rodríguez-Liñán C. (2017). Eco-efficient acoustic and thermal conditioning using false ceiling plates made from plaster and wood waste. J. Clean. Prod..

[18] Cherki A.-B., Remy B., Khabbazi A., Jannot Y., Baillis D. (2014). Experimental thermal properties characterization of insulating cork–gypsum composite. Constr. Build. Mater..

[19] Liuzzi S., Rubino C., Stefanizzi P. (2017). Use of clay and olive pruning waste for building materials with high hygrothermal performances. Energy Procedia.

[20] Sánchez Guerrero C.M., Chávez Valencia L.E. (2017). UG Rock. Jóvenes Cienc..

[21] Rossetto J.R.D.M., Correia L.S., Geraldo R.H., Camarini G. (2015). Gypsum Plaster Waste Recycling: Analysis of Calcination Time. Key Eng. Mater..

[22] Erbs A., Nagalli A., De Carvalho K.Q., Mymrin V., Passig F.H., Mazer W. (2018). Properties of recycled gypsum from gypsum plasterboards and commercial gypsum throughout recycling cycles. J. Clean. Prod..

[23] Pinheiro S.M.M., Camarini G. (2015). Characteristics of Gypsum Recycling in Different Cycles. Int. J. Eng. Technol..

[24] Pedreño-Rojas M., Flores-Colen I., De Brito J., Rodríguez-Liñán C. (2019). Influence of the heating process on the use of gypsum wastes in plasters: Mechanical, thermal and environmental analysis. J. Clean. Prod..

[25] Rivero A.J., Sathre R., Navarro J.G. (2016). Life cycle energy and material flow implications of gypsum plasterboard recycling in the European Union. Resour. Conserv. Recycl..

[26] Gómez-Rojo R., Alameda L., Rodríguez Á., Calderón V., Gutiérrez-González S. (2019). Characterization of Polyurethane Foam Waste for Reuse in Eco-Efficient Building Materials. Polymers.

[27] Khalil A., Tawfik A., Hegazy A., El-Shahat M. (2014). Effect of some waste additives on the physical and mechanical properties of gypsum plaster composites. Constr. Build. Mater..

[28] Zhu C., Zhang J., Peng J., Cao W., Liu J. (2018). Physical and mechanical properties of gypsum-based composites reinforced with PVA and PP fibers. Constr. Build. Mater..

[29] UNE-EN 13279-1:2009 (2009). Construction Plasters and Gypsum-Based Binders for Construction. Part 1: Definitions and Specifications.

[30] UNE-EN 13279-2:2014 (2014). Construction Plasters and Gypsum-Based Binders for Construction. Part 2: Test Methods.

[31] UNE-EN 520:2005+A1 (2010). Sheetrock Plasterboard. Definitions, Specifications and Testing Methods.

[32] UNE 102042 (2014). Construction Plasters. Other Test Methods.

[33] UNE-EN 12667:2002 (2002). Construction Materials. Determination of Thermal Resistance by the Stored Hot Plate Method and the Heat Flow Meter Method. Products with High and Medium Thermal Resistance.

[34] De Villanueva Domínguez L., García Santos A. (2001). Manual del yeso. Asociación Técnica y Empresarial del Yeso.

[35] CTE (2006). Código Técnico de la Edificación.

[36] Del Cura S.H. (2016). Influencia de la Dosificación y Granulometría del Caucho de Neumático Fuera de uso (NFU) y de las Dimensiones Físicas en las Propiedades Térmicas, Acústicas y Mecánicas de Placas de Mortero de Yeso y Caucho. Ph.D. Thesis.

[37] Domínguez Lepe J., Guemez Pacheco D. (2011). Fabricación y evaluación de paneles aplicables a la industria de la construcción a partir del reciclaje de envases multicapa. Ingeniería.

[38] Santos A.G. (2004). Characterization of reinforced scagliola compounds, related to the type of reinforcement and W/P ratio. Inf. Construcción.

[39] Bicer A., Kar F. (2017). Thermal and mechanical properties of gypsum plaster mixed with expanded polystyrene and tragacanth. Therm. Sci. Eng. Prog..

[40] Mayor Lobo P., Bustamante Montoro R., Rangel C., Hernández Olivares F. (2008). Propiedades térmicas, acústicas y mecánicas de placas de mortero de yeso-caucho. Actas de II Jornadas de Investigación en Construcción.

[41] Erdem S., Arioğlu N. (2017). An Analysis of the Properties of Recycled PET Fiber-Gypsum Composites. A/Z ITU J. Fac. Arch..

[42] Barriguete A.V., Merino M.D.R., Sánchez E.A., Ramírez C.P., Arrebola C.V. (2018). Analysis of the feasibility of the use of CDW as a low-environmental-impact aggregate in conglomerates. Constr. Build. Mater..

[43] Pedreño-Rojas M.A., Rodríguez-Liñán C., Flores-Colen I., De Brito J. (2020). Use of Polycarbonate Waste as Aggregate in Recycled Gypsum Plasters. Materials.

[44] Vidales-Barriguete A., Atanes-Sánchez E., del Río-Merino M., Piña-Ramírez C. (2020). Analysis of the improved water-resistant properties of plaster compounds with the addition of plastic waste. Constr. Build. Mater..

[45] Instituto de Ciencias de la Construcción Eduardo Torroja. CSIC Código Técnico de la Edificación—CTE. https://www.codigotecnico.org/index.html.

[46] Vidales-Barriguete A., Piña-Ramírez C., Serrano-Somolinos R., del Río-Merino M., Atanes-Sánchez E. (2021). Behavior resulting from fire in plasterboard with plastic cable waste aggregates. J. Build. Eng..

[47] Diez R.V.L., Zaldivar O.L., DEL Cura S.H., Lobo P.L.M., Olivares F.H. (2019). Influencia de la incorporación de fibras de caucho procedente de neumáticos fuera de uso (NFU) en morteros de yeso. Estudio de las propiedades mecánicas, térmicas y acústicas. DYNA Ing. E Ind..

[48] Abu-Lebdeh T., Fini E., Fadiel A. (2014). Thermal conductivity of rubberized gypsum board. Am. J. Eng. Appl. Sci..

[49] Sahin S., Karaman S. (2012). The Properties of Expanded Polystyrene—Pumice—Gypsum Blocks as a Building Material. J. Tekirdag Agric. Fac..

